# Examining the psychometric properties of the Norwegian version of the Social Aptitudes Scale in two clinical samples

**DOI:** 10.1186/s40359-023-01258-4

**Published:** 2023-08-03

**Authors:** Sabine Kaiser, Børge Mathiassen, Bjørn Helge Handegård, Yngvild Arnesen, Marianne Berg Halvorsen

**Affiliations:** 1 Regional Center for Child and Youth Mental Health - North, UiT The Arctic University of Norway, Tromsø, Norway; 2 Department of Child and Adolescent Psychiatry, University Hospital of North Norway, Tromsø, Norway; 3 Department of Psychology, UiT The Arctic University of Norway, Tromsø, Norway; 4 Department of Pediatric Rehabilitation, University Hospital of North Norway, Tromsø, Norway

**Keywords:** Social Aptitudes Scale, Psychometric properties, Autism spectrum disorder, Neurodevelopmental disorders, Child and adolescent’s mental health services

## Abstract

**Background:**

Few studies have examined the psychometric properties of the Social Aptitudes Scale (SAS). The study aims of the current paper were to examine the internal consistency and the validity of the Norwegian SAS.

**Methods:**

Parents of children from a clinical neuropediatric sample (*N* = 257) and from a clinical sample from child and adolescent’s mental health services (*N* = 804) filled in the SAS.

**Results:**

Internal consistency for the SAS were good in both samples and correlations between the SAS and different scales were in the expected directions. The results from the Confirmatory Factor Analyses indicated poor model fit.

**Conclusions:**

Future validity studies should investigate whether SAS is suitable as a screening instrument for detecting autism spectrum disorder.

## Introduction

Social skills are skills that an individual exhibits when communicating and interacting with others. It includes all verbal- and non-verbal communication, and skills like sharing, cooperating, being empathetic, and listening to what other people have to say. Social skills are important for the mental well-being and in order to build and maintain good relationships [1]. Difficulties in social skills are associated with many mental disorders [2, 3], including neurodevelopmental disorders. Autism spectrum disorder (ASD) is, for example, characterized by persistent deficits in communication and interaction, including deficits in social-emotional reciprocity, in nonverbal communication, and in developing, understanding, and maintaining relationships, in addition to restricted and repetitive behaviors, activities, or interests [4].

The Social Aptitudes Scale (SAS) is a ten-item questionnaire that measures skills in social understanding and behavior that vary from individual to individual and that are usually not fully developed in ASD [5]. Each item is rated on a on a five-point scale from 0 (A lot worse than average), 1 (A bit worse than average), 2 (About average), 3 (A bit better than average), and 4 (A lot better than average), with lower scores indicating worse function – the reference group being other children of the same age. The SAS is part of the Development and Well-Being Assessment (DAWBA; [6]). The items focus on interactive skills (e.g., “Able to compromise and be flexible" and “Easy to chat with, even if it isn’t on a topic that specially interests him/her”) rather than on relatively easily learned skills (e.g., saying “please” or “thank you”; [5]). The SAS is used together with the ASD diagnosis module to diagnose ASD in the DAWBA. That is, a score of 12 or less on the SAS indicated difficulties in social functioning and necessitates that all items of the ASD diagnostic section of the DAWBA are completed.

There are only two studies who report on the psychometric properties of the SAS [5, 7]. The first study that presented and examined the SAS was conducted by Liddle, Batty [5] in a large and representative community sample of British parents of 5–16-year-old children and adolescents. They report that all items of the SAS loaded on one factor and calculated a Cronbach’s alpha of 0.88 for the SAS. Liddle, Batty [5] presented the distribution of the total SAS scores for three age groups and found that the modal score was 20, which represents a mean item score of 2. Correlations between scores from the SAS and the Strengths and Difficulties Questionnaire (SDQ; [8]) were in the expected directions, and correlations were low to moderate indicating that the two instruments assess different constructs. The strongest correlations were found between the SAS and the SDQ total difficulties score (*r* =  − 0.44, *p* < 0.001, *N* = 7758) and the SDQ prosocial scale (*r* = 0.42, *p* < 0.001, *N* = 7758) both from the SDQ parent version, respectively. The SAS was better at discriminating between children with and without ASD than the SDQ total score [5]. Liddle, Batty [5] concluded that children and adolescents “with low SAS scores are at an increased risk of mental health problems, with ASDs becoming particularly likely at very low scores” (p. 513).

The other study that examined the SAS was conducted by Axelrud, DeSousa [7] in a relatively large sample of Brazilian parents of 6–14-year-old children and adolescents from a “high-risk study for psychiatric disorders”. Fit indices from the confirmatory factor analysis (CFA) indicated good model fit for the one-factor model of the SAS. The correlation between the SAS and the Child Behavior Checklist social problems scale was in the expected direction and was found to be higher for children and adolescents with low scores (i.e., worse functioning) on the SAS (*r* = -0.52, *p* < 0.001) and non-significant for children and adolescents with high scores on the SAS (*r* = -0.05, *p* = 0.65). The SAS predicted psychiatric disorders and the number of friends and, to be more specific, diagnoses like ASD, ADHD, and conduct disorder. The authors concluded that the results of their study “provide further validity to the SAS as an appropriate measure of social aptitudes in the population” ([7]; p. 1038).

Also, few other studies have used the SAS in research (e.g., [9–12]). Benarous, Mikita [12] found that lower SAS scores were related to higher parent-reported manic symptoms compared to the general population. Kochhar, Batty [9] found that children and adolescents with attention deficit hyperactivity disorder (ADHD) had lower SAS scores than a control group. Similarly, Rhind, Bonfioli [11] found that adolescents with anorexia nervosa had lower scores on the SAS compared to a healthy population. Cronbach’s alpha was excellent in their study. Maruyama, Santos [10] used the SAS and found that maternal depression affected their children’s social aptitudes in adolescents. Cronbach’s alpha was adequate in their study.

### Study aims

The SAS is a potentially time-saving questionnaire of social functioning in the clinic as it only consists of 10-items. However, the SAS's strengths and weaknesses are poorly elucidated as very few studies have examined its psychometric properties, and none of them in clinical samples. Accordingly, the aims of the present study are to examine the psychometric properties of the Norwegian version of the SAS in two clinical samples of children and adolescents, one neuropediatric sample and one sample recruited from child and adolescent’s mental health services (CAMHS). We will examine the reliability of the SAS, correlations of the SAS with the SDQ, and the factor structure of the SAS. We expected the strongest and positive correlations with the SDQ Prosocial scale and the strongest negative correlations with the SDQ Peer problem scale. In the neuropediatric sample we will, furthermore, examine correlations with the Vineland Adaptive Behavior Scales 2^nd^ edition (VABS-II) social skills and communication scales and the scales and total score from the Social Responsiveness Scale (SRS). This cannot be done in the CAMHS sample, since we only have VABS-II and SRS scores from the neuropediatric sample.

## Methods

### Participants

Two clinical samples were included in the study. The first one is a clinical neuropediatric sample of 257 children and adolescents that were referred for a developmental and neurological assessment to the neuropediatric outpatient clinics at the University Hospital of North Norway (UNN; *n* = 216) and the Finnmark Hospital Trust (FIN; *n* = 41). Children under four years were excluded from the study because of a lack of suitability of at least one of the instruments used in the study. Another exclusion criterion was a lack of parental fluency in Norwegian. The most frequent neurodevelopmental disorders in the sample were (a) specific developmental disorders (33.1%), (b) intellectual disability (ID; 18.7%, none with severe IDs), (c) other diseases of the nervous system such as epilepsy and cerebral palsy (17.9%), (d) ASD (13.6%), (e) ADHD (13.6%), and (f) congenital malformations and chromosomal abnormalities (10.5%). Specific developmental disorder was operationalized according to ICD-10. This included F80 specific developmental disorders of speech and language, F81 specific developmental disorders of scholastic skills, F82 specific developmental disorder of motor function, and F 83 mixed specific developmental disorders. A given subject could have more than one diagnosis. For further description of the design and samples, see Halvorsen, Aman [13], Halvorsen, Mathiassen [14], or Halvorsen, Mathiassen [15].

The second clinical sample includes 804 patients from the Child and Adolescents Mental Health Services at UNN. UNN serves as a specialist health care hospital a population of 190,726 residents of the county municipalities of Troms and the northern part of Nordland. The health care trust is covering an area of approximately 31,300 km^2^. Annually, UNN provides mental health services to about 5% (2100/42,000) of the population aged 0–18 years. About 60% of the treated patients are new referrals from general practitioners and the child protection services. The CAMHS at UNN consist of six outpatient and one inpatient clinic. All of them include the online version of the DAWBA [6] in the routine clinical assessment. The questionnaires SAS and SDQ are an integrated part of DAWBA. The main diagnostic groupings based on the DAWBA in the sample were, in descending order: (a) emotional disorders (57.6%), (b) behavioral disorders (47.3%), (c) hyperkinesis (28.1%), and (d) ASD (2.6%). All DAWBA data at UNN are stored in a de-identified local CAMHS quality register. For further description of the design and sample, see Fernández de la Cruz, Vidal-Ribas [16]. The data protection officer at UNN has approved the use of data from the quality register for research purposes.

The main demographic and clinical characteristics of the neuropediatric- and the CAMHS sample can be found in Table 1. The mean age of the neuropediatric sample was 10.54 years (*SD* = 3.46; range 5 to 18 years) and 11.88 years (*SD* = 3.52; range 4 to 19 years) in the CAMHS sample.

**Table 1:** Descriptive Statistics and Bivariate Relationships between the SAS and Demographic and Clinical Variables in the Neuropediatric Sample (*N* = 257) and the CAMHS Sample (*N* = 804)

	Neuropediatric sample		CAMHS sample	
Variable	*M* (*SD*)/ *n* (%)	SAS total *r*	*M* (*SD*)/ *n* (%)	SAS total *r*
Age	10.54 (3.46)	− .15**	11.88 (3.52)	.17***
Gender boys^a^	165 (64.2%)	.10	423 (52.6%)	− .19***
SDQ Emotional	3.35 (2.64)	− .29***	4.61 (2.77)	− .06
SDQ Behavioral	2.01 (1.93)	− .45***	2.90 (2.15)	− .42***
SDQ Hyperactivity	5.14 (2.59)	− .35***	5.03 (2.78)	− .36***
SDQ Peer problem	3.75 (2.52)	− .50***	3.20 (2.31)	− .32***
SDQ Prosocial	7.25 (2.35)	.62***	7.06 (2.27)	.54***
SDQ Total	14.25 (6.97)	− .55***	15.74 (6.47)	− .44***
SDQ Impact	3.40 (2.78)	− .48***	4.18 (2.75)	− .27***
VABS − II Socialization	74.20 (15.33)	.56***	−	−
VABS − II Communication	65.89 (13.89)	.41***	−	−
VABS − II Total	68.70 (14.88)	.52***	−	−
SRS Social Awareness	8.33 (4.01)	− .62***	−	−
SRS Social Cognition	11.18 (6.67)	− .62***	−	−
SRS Social Communication	19.28 (10.72)	− .63***	−	−
SRS Social Motivation	11.46 (5.91)	− .46***	−	−
SRS Autistic Mannerisms	9.09 (6.59)	− .52***	−	−
SRS Total	59.43 (30.13)	− .65***	−	−
ASD Status^b^	29 (11.3%)	− .20***	−	−
ADHD Status^c^	28 (10.9%)	− .14*	−	−
ID Status^d^	39 (15.2%)	− .13*	−	−

*CAMHS sample* Child and Adolescents Mental Health Service sample, *ASD* Autism Spectrum Disorder (without ADHD or ID), *ADHD* Attention Deficit Hyperactivity Disorder (without ASD or ID), *ID* Intellectual Disability (without ASD or ADHD). *SDQ* Strengths and Difficulties Questionnaire, *VABS − II* Vineland Adaptive Behavior Scales, *SRS* Social Responsiveness Scale

^a^Gender: 1 = boy and 0 = girl

^b^ASD Status: 1 = present and 0 = absence

^c^ADHD status: 1 = present and 0 = absence

^d^ID Status: 1 = present and 0 = absence

*p < .05

**p < .01

***p < .001

### Measures

*The Norwegian SAS* [5] was used to assess social skills of the children and adolescents and the form was completed by their parents. The SAS is a ten-item questionnaire (e.g., “Aware of what is and is not socially appropriate”) and each item is rated on a on a five-point scale from 0 (A lot worse than average) through 4 (A lot better than average), with lower scores indicating worse function. Detailed information about SAS can be found elsewhere (http://dawba.info/SAS/). Both the neuropediatric- and the CAMHS sample completed the SAS in the DAWBA.

*The Norwegian Social Responsiveness Scale* [17] is a parent-completed screening questionnaire often used to measure ASD severity. It is composed of 65 items within the five subdomains Social Awareness, Social Cognition, Social Communication, Social Motivation, and Autistics Mannerisms in addition to an overall total score. Parents respond to how often their child displays a given behavior on a four-point Likert scale from 0 (not true) through 3 (almost always true) in the past six months, with higher scores indicating worse function. The manual recommends the use of the SRS raw scores in research. The SRS has been validated in different cultures, with results indicating good psychometric properties (e.g., [18]), and scores on the SRS are strongly correlated with Autism Diagnostic Interview – Revised domain scores (*r* = 0.65–0.77; [19]). Only the neuropediatric sample completed the SRS and Cronbach’s alpha ranged from 0.69 (Social Awareness) to 0.89 (Social Communication). SRS scores were missing for 14 children.

*The Norwegian SDQ* parent version [8] was used in the current study. The SDQ is a 25-item mental health questionnaire covering four problem areas (emotional, hyperactivity-inattention, conduct, and peer problems), one area of strength (prosocial behavior), and additional questions related to distress and functional impairment. Each item is rated on a three-point scale from 0 (not true) through 2 (certainly true). The SDQ has been validated in different cultures, with results indicating good psychometric properties [20, 21]. Both the neuropediatric- and the CAMHS samples completed the SDQ in the DAWBA. In the neuropediatric sample Cronbach’s alpha ranged from 0.69 (conduct) to 0.80 (prosocial behavior) for the parent version. In the CAMHS sample Cronbach’s alpha ranged from 0.65 (peer problem) to 0.81 (hyperactivity-inattention) for the parent version.

*The Norwegian version of the DAWBA* [6] was used to establish diagnoses in the CAMHS sample based on DSM-IV diagnostic criteria [22]. The DAWBA is a detailed diagnostic tool completed by parents (takes approximately 30 min), and youths (takes approximately 10 min), with a briefer questionnaire for teachers (takes approximately 10 min) In the current study, the DAWBA was completed as an online package of questionnaires on admission to the clinics. In this paper, we group mental disorders into emotional disorder (including anxiety and depressive disorders); behavioral disorders (including oppositional defiant and conduct disorders); ADHD; and ASD (including autism and Asperger’s syndrome). Participants were assigned a positive diagnosis if they scored 3 or higher in the relevant DAWBA bands [23], as previously described [16]. The DAWBA has shown good discriminative ability in both population-based samples and clinical samples, as well as across different categories of diagnoses [6]. Both in Norway and Great Britain, the DAWBA generates realistic estimates of prevalence for psychiatric illnesses as well as high predictive validity when used in public health services [24, 25]. As a SAS score of 12 or less are included as part of the diagnostic process towards an ASD diagnosis in the DAWBA, we limited the examination of SAS psychometric properties in the CAMHS sample to factor structure and correlations of SAS and SDQ scores, and not relation to an ASD diagnosis, to avoid circularity. Detailed information about SAS can be found elsewhere (www.dawba.info). We report DAWBA data for the CAMHS sample.

*VABS-II* [26], a semi-structured interview, was used to establish the child’s adaptive level of functioning and includes the following four domains with related subdomains: Communication (receptive, expressive, and written), Daily Living Skills (personal, domestic, and community), Socialization (interpersonal relationships, play and leisure time, and coping skills), and Motor Skills (gross and fine). The neuropediatric sample completed the VABS-II and the VABS-II total, the Communication, and the Socialization scores were used. VABS-II scores were missing for 15 children.

### Procedure

*Neuropediatric sample:* The children underwent an interdisciplinary, neurodevelopmental/neurological assessment over two days, where they were assessed for the presence of a neurological/ neurodevelopmental disorder. In addition, the examinations included MRI Caput, EEG and/or genetic testing. Clinical psychologist/ neuropsychologist administered the VABS-II and a standardized intelligence scale. Diagnoses were based on ICD-10 criteria [27, 28]. A score below 70 on both the standardized intelligence test and the VABS-II was used to diagnose the presence of an ID. Furthermore, in the current study, ASD diagnoses for the neuropediatric sample were not based on the computer-predictions from the DAWBA, but on clinic diagnoses where the results from the ADI-R and the Autism Diagnostic Observation Schedule often were included in the assessments.

*CAMHS sample:* Parents, children and their teachers complete the online version of the DAWBA [6] in the routine clinical assessment. The questionnaires SAS and SDQ are an integrated part of DAWBA. The data protection officer at UNN allowed to analyze the two datasets for the neuropediatric- and the CAMHS sample separately, but did not agree to merge the two files to one data file.

### Statistical analysis

The statistical analyses were conducted with SPSS and included the calculation of Pearson’ correlations (*r*), McDonald’s Omega, and McDonald’s Omega if item deleted. The guidelines from the European Federation of Psychologists’ Association (EFPA; [29]) for the evaluation of the psychometric properties were used. To evaluate congruent validity correlations of *r* < 0.55 are considered inadequate, *r* between 0.55 and 0.64 as adequate, *r* between 0.65 and 0.74 as good, and *r* of 0.75 or bigger as excellent. To evaluate McDonald’s Omega coefficients smaller than 0.70 are considered inadequate, coefficients between 0.70 and 0.79 as adequate, coefficients between 0.80 and 0.89 as good, and coefficients of 0.90 or bigger as excellent [29].

A CFA-model with one general latent factor with ten indicators from the SAS was tested separately for each sample, respectively, using Mplus. The weighted least square mean and variance adjusted (WLSMV) estimator was used. Different fit indices were used to evaluate model fit: The χ^2^-statistic, the χ^2^/degrees of freedom ratio (χ^2^/df) with a threshold level of 3.00 or 2.00, the Comparative Fit Index (CFI) and the Tucker Lewis Index (TLI), where greater values than 0.90 or 0.95 indicate good model fit, and the Root Mean Square Error of Approximation (RMSEA), where smaller values than 0.07 or 0.06 indicate good model fit [30].

## Results

The mean SAS scores for the neuropediatric- and CAMHS sample were *M* = 14.16 (*SD* = 5.89; *N* = 257) and *M* = 17.77 (*SD* = 5.67; *N* = 804), respectively.

### Reliability

McDonald’s Omega for the SAS were good with coefficients of 0.88 and 0.87 in the neuropediatric- and CAMHS sample, respectively. Deleting an item from the SAS for both samples would, in most cases, have decreased the McDonald’s Omega (Table 2). For example, in the CAMHS sample McDonald’s Omega would be 0.845 when deleting item 7 “Can tell what others think and feel”.

**Table 2:** McDonald’s Omega if Item Deleted for the SAS in the Neuropediatric Sample (*N* = 257) and the CAMHS Sample (*N* = 804)

		McDonald’s Omega if item deleted	
SAS item		Neuropediatric sample	CAMHS sample
1	Can laugh around with others	.87	.87
2	Easy to chat with	.87	.86
3	Flexible, can compromise	.86	.85
4	Can defuse tense situations	.86	.85
5	Good loser	.88	.87
6	Puts others at ease	.87	.86
7	Can tell what others think and feel	.86	.85
8	Apologizes, puts things right	.86	.86
9	Leads without seeming bossy	.86	.86
10	Recognizes what is socially appropriate	.86	.85

*CAMHS* Child and Adolescents Mental Health Service

### Relationship between the SAS and clinical variables

In the neuropediatric sample, the strongest correlation between the SAS and the SDQ were found for the SDQ Prosocial behavior scale (*r* = 0.62, *p* < 0.001) followed by the SDQ total difficulties score (*r* =  − 0.55, *p* < 0.001; see Table 1). Likewise, in the CAMHS sample the strongest correlation between the SAS and the SDQ were found for the SDQ Prosocial behavior scale (*r* = 0.54, *p* < 0.001) followed by the SDQ total difficulties score (*r* =  − 0.44, *p* < 0.001).

Furthermore, for the neuropediatric sample, the strongest correlation between the SAS and the remaining instruments were found for the SAS and the SRS Total score (*r* =  − 0.65, *p* < 0.001), the SRS Social Communication scale (*r* =  − 0.63, *p* < 0.001) and SRS Social Awareness and SRS Social Cognition scale (*r* =  − 0.62, *p* < 0.001), respectively. The correlation between the SAS and the VABS − II Socialization scale was *r* = 0.56 (*p* < 0.001).

### Confirmatory factor analyses results

**Fig. 1:**
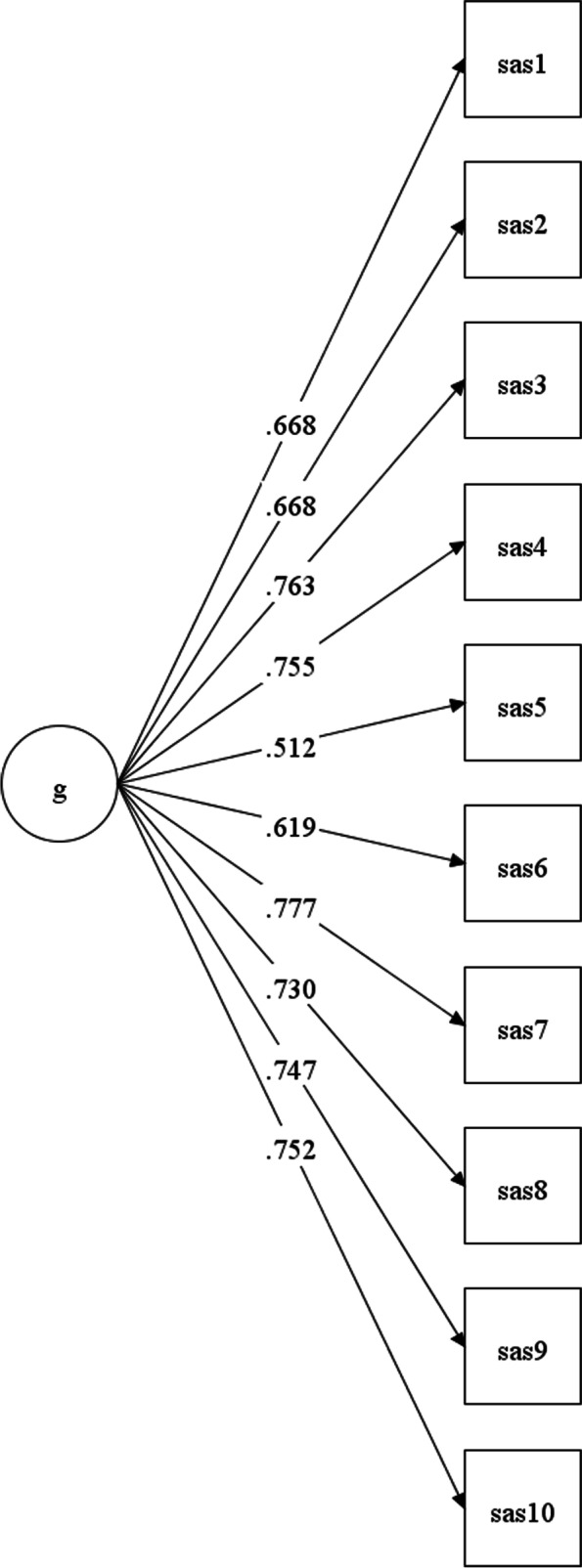
Presents the standardized factor loadings for the ten items of the SAS for the neuropediatric sample

Two CFAs were conducted to examine the one-factor model of the SAS in the neuropediatric and the CAMHS sample, respectively. The fit indices for the CFA of the SAS in the neuropediatric sample were as follows: The χ^2^(35, *N* = 257) was 153.25 (*p* < 0.001; χ^2^/df = 4.38), the CFI was 0.953, the TLI was 0.940, and the RMSEA was 0.115 (90% CI [0.096, 0.134]). The standardized factor loadings for the ten items ranged from 0.51 (item 5: “Graceful when s/he doesn’t win or get his/her own way. A good loser.”) to 0.78 (item 7: “By reading between the lines of what people say, s/he can work out what they are really thinking and feeling.”; Fig. 1).

**Fig. 2:**
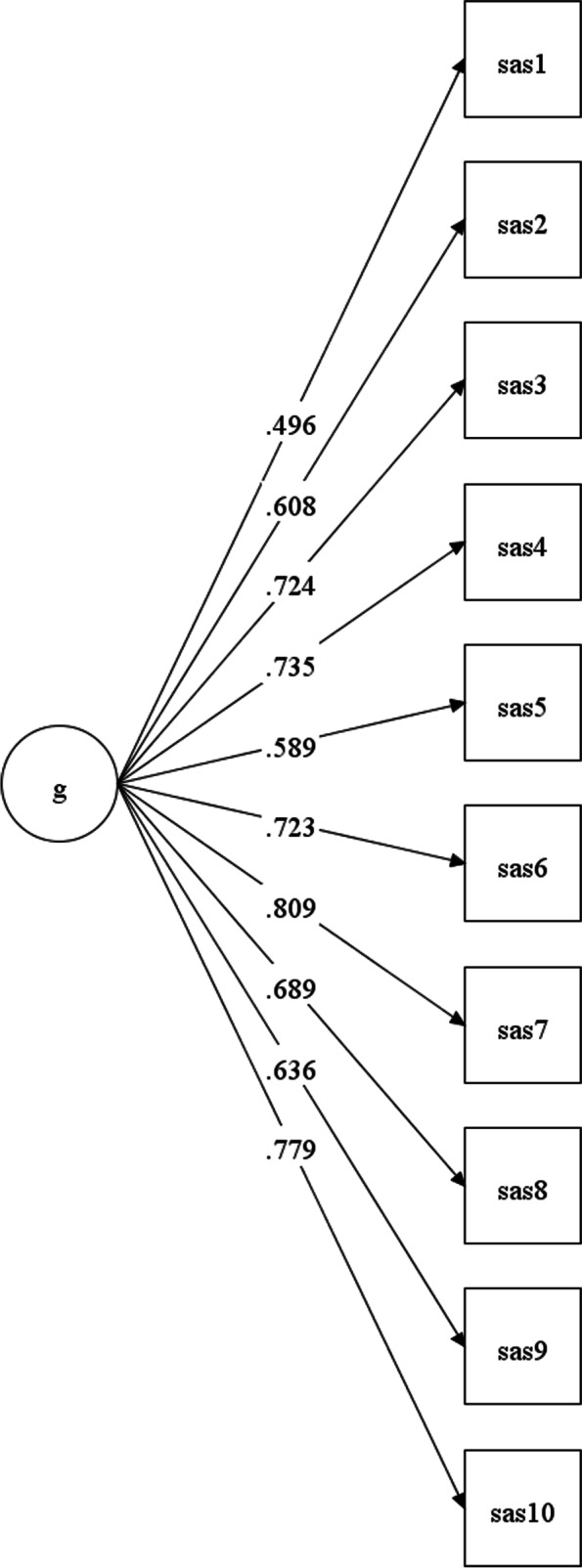
Presents the standardized factor loadings for the ten items of the SAS for the CAMHS sample

The fit indices for the CFA of the SAS in the CAMHS sample were as follows: The χ^2^(35, *N* = 804) was 254.74 (*p* < 0.001; χ^2^/df = 7.28), the CFI was 0.968, the TLI was 0.959, and the RMSEA was 0.088 (90% CI [0.078, 0.099]). The standardized factor loadings for the ten items ranged from 0.50 (item 1: “Able to laugh around with others, for example accepting light-hearted teasing and responding appropriately.”) to 0.81 (item 7: “By reading between the lines of what people say, s/he can work out what they are really thinking and feeling.”; Fig. 2).

## Discussion

The aims of the present study were to examine the psychometric properties of the Norwegian SAS in two clinical samples of children and adolescents. McDonalds Omega for the SAS were good in both samples [29] and in accordance to findings from Liddle, Batty [5] and Rhind, Bonfioli [11].

In both samples, the strongest correlation between the SAS and SDQ scales were found for the SDQ Prosocial behavior scale. Correlations were adequate in accordance with the guidelines from the EFPA manual, indicating that the two instruments measure similar but not identical constructs [29]. Also, in the Liddle, Batty [5] study the highest correlations were found between the SAS and the SDQ Prosocial behavior scale parent version, but inadequate (i.e., *r* < 0.55) in relation to the evaluation of convergent validity. In the current study, the SDQ Peer problem scale correlated also inadequately with the SAS in both samples. Liddle, Batty [5] reported even lower correlations and that the results supported “the conceptual distinction between social aptitude and peer relationships […] good social aptitudes do not guarantee good peer relationships, and neither do poor social aptitudes preclude good peer relationships” (p. 512). However, we believe that it is of interest to look at the association between the SAS and these two SDQ dimensions (i.e., the SDQ Peer problem scale and the SDQ Prosocial scale) in order to elucidating convergent validity, because it is likely that prosocial behavior is associated with social aptitude. As such, individuals who are perceived as less social are probably also perceived as less prosocial and thus, should score higher on the SDQ Peer problem scale. Individuals with challenges with social aptitude are expected to have more friend problems and less prosocial behavior. However, the findings of the current study indicate convergent validity between the SAS and the SDQ Prosocial behavior scale but not with the SDQ Peer problem scale.

For the neuropediatric sample, the strongest correlation between the SAS and the remaining instruments were found for the SAS and the SRS Total score, the SRS Social Communication scale, SRS Social Awareness, and SRS Social Cognition scale. Correlations were adequate, indicating that the two instruments measure similar but not identical constructs (EFPA; [29]). Correlations between the SAS and the VABS-II Socialization and Communication scales were in the expected directions but not as strong as between the SAS and the SRS.

The fit indices of the CFAs indicate mixed results. Although the CFI and TLI were high in both analyses, indicating good model fit, the RMSEA were also high, indicating poor model fit. The reason for this is that high correlations between the questions within one factor quickly can give a high CFI and TLI, but variation in the correlations between the factors can give a weak model-fit according to the RMSEA. In conclusion, however, the results of the CFAs in the present study indicate that the data do not fit the proposed model well. This is not in accordance to the results of Axelrud, DeSousa [7], who found good model fit in a “high-risk study for psychiatric disorders” (p. 1032) in a relatively large sample of children recruited from public schools in two Brazilian cities. One reason may be that when parents rate their child on the ten statements of the SAS, they are instructed to compare their child with other children of the same age (the instruction is “How does [Name] compare with other children/people of his/her age in the following situations:”). Different respondents or parents will not necessarily rate their child to the same comparison group, that is, is the comparison group a patient group who might have similar difficulties in social interaction as their child, or is the comparison group a “healthy” group with possible better social skills [5]. Furthermore, and although it is an advantage of the SAS that it is a short questionnaire, it uses double-barreled items, that is statements which focus on more than one topic (e.g., “Able to laugh around with others, for example accepting light-hearted teasing and responding appropriately” or “Easy to chat with, even if it isn’t on a topic that specially interests him/her.”). These complex items might lead to challenges when filling out the questionnaire. Reformulating the statements so that they only touch one topic at a time would be one possibility to improve the SAS.

### Strengths and limitations

A strong point of this study is that the psychometric properties of the SAS were examined in two clinical samples of children and adolescents. Both samples were from services from the largest university hospital in Northern Norway. The inclusion of two clinical samples can result in small variation and this in turn to low correlations for example between the SAS and other variables. In the current sample, however, the variation in SAS scores was good. Furthermore, it has to be taken into account that the SAS and the SDQ were filled in by the parents of the children or adolescents in both samples, which might partly explain why the correlations between these instruments were relatively strong (shared rater effect). It is also weakness of SAS that it only uses parental reporting and that it does not collect information from multiple informants such as teachers to get a more complete picture of a child. Also, both the SAS and the SDQ were developed by the same person, and it is perhaps not so strange that the correlations become "acceptable" when examining convergent validity.

## Conclusion

The results of the present study, which examined the SAS’s psychometric properties for the first time in specialist clinics, suggest that the Norwegian version of the SAS has good reliability and acceptable convergent validity as it overlaps meaningfully with other established measures tapping social functioning (i.e., with the SDQ Prosocial behavior scale, the SRS, and the VABS-II Socialization domain). The results of the factor analyses, on the other hand, revealed no good model-fit indicating problems with the factor structure. Further validity studies and refinement of the scale in clinical samples is therefore recommended. Also, it remains to be investigated how the SAS functions as a screening instrument on its own for ASD in specialist clinics. Future studies using direct head-to-head comparison of different ASD screening measures would be instructive.

## Data Availability

The datasets analyzed during the current study are not publicly available due to ethical restrictions and personal data protection but are available from the authors on reasonable request and with permission of the Data Protection Official in the health trusts.
